# Disproportionate Mitral Regurgitation Determines Survival in Acute Heart Failure

**DOI:** 10.3389/fcvm.2021.742224

**Published:** 2021-12-02

**Authors:** Max Berrill, Ian Beeton, David Fluck, Isaac John, Otar Lazariashvili, Jack Stewart, Eshan Ashcroft, Jonathan Belsey, Pankaj Sharma, Aigul Baltabaeva

**Affiliations:** ^1^Department of Cardiology, St. Peter's Hospital, Surrey, United Kingdom; ^2^Department of Research and Development, St. Peter's Hospital, Surrey, United Kingdom; ^3^Institute of Cardiovascular Research, Royal Holloway University, University of London, Egham, United Kingdom; ^4^JB Medical Ltd., Sudbury, United Kingdom; ^5^Department of Cardiology, Royal Brompton and Harefield Hospital, London, United Kingdom

**Keywords:** acute heart failure (AHF), mitral regurgitation, disproportionate mitral regurgitation, heart failure, disproportionate MR, disproportionate

## Abstract

**Objectives:** To assess the prevalence and impact of mitral regurgitation (MR) on survival in patients presenting to hospital in acute heart failure (AHF) using traditional echocardiographic assessment alongside more novel indices of proportionality.

**Background:** It remains unclear if the severity of MR plays a significant role in determining outcomes in AHF. There is also uncertainty as to the clinical relevance of indexing MR to left ventricular volumes. This concept of disproportionality has not been assessed in AHF.

**Methods:** A total of 418 consecutive patients presenting in AHF over 12 months were recruited and followed up for 2 years. MR was quantitatively assessed within 24 h of recruitment. Standard proximal isovelocity surface area (PISA) and a novel proportionality index of effective regurgitant orifice/left ventricular end-diastolic volume (ERO/LVEDV) >0.14 mm^2^/ml were used to identify severe and disproportionate MR.

**Results:** Every patient had MR. About 331/418 (78.9%) patients were quantifiable by PISA. About 165/418 (39.5%) patients displayed significant MR. A larger cohort displayed disproportionate MR defined by either a proportionality index using ERO/LVEDV > 0.14 mm^2^/ml or regurgitant volumes/LVEDV > 0.2 [217/331 (65.6%) and 222/345 (64.3%), respectively]. The LVEDV was enlarged in significant MR−129.5 ± 58.95 vs. 100.0 ± 49.91 ml in mild, [*p* < 0.0001], but remained within the normal range. Significant MR was associated with a greater mortality at 2 years {44.2 vs. 34.8% in mild MR [hazard ratio (HR) 1.39; 95% CI: 1.01–1.92, *p* = 0.04]}, which persisted with adjustment for comorbid conditions (HR; 1.43; 95% CI: 1.04–1.97, *p* = 0.03). Disproportionate MR defined by ERO/LVEDV >0.14 mm^2^/ml was also associated with worse outcome [42.4 vs. 28.3% (HR 1.62; 95% CI 1.12–2.34, *p* = 0.01)].

**Conclusions:** MR was a universal feature in AHF and determines outcome in significant cases. Furthermore, disproportionate MR, defined either by effective regurgitant orifice (ERO) or volumetrically, is associated with a worse prognosis despite the absence of adverse left ventricular (LV) remodeling. These findings outline the importance of adjusting acute volume overload to LV volumes and call for a review of the current standards of MR assessment.

**Clinical Trial Registration:** https://clinicaltrials.gov/ct2/show/NCT02728739, identifier NCT02728739.

## Introduction

Acute heart failure (AHF) is associated with high mortality (1) and remains a substantial financial and healthcare burden (2). The recognition and prevention of precipitating factors, therefore, remain of the utmost importance (3). Acute and worsening of chronic degenerative mitral regurgitation (MR) (4, 5) is a recognized cause of AHF-related hospitalization (6) whereas the role of functional mitral regurgitation (FMR), secondary to cardiac remodeling and left ventricular (LV) dysfunction (7), is less established.

Functional mitral regurgitation (FMR) has a significant impact on morbidity and mortality (8). However, the complexity and heterogeneity of myocardial disease in heart failure (HF) and the subsequent alterations to the mitral valve apparatus have made the quantitative analysis of MR difficult. This has created disagreements between guidelines that suggest differing cut-offs for severe FMR (9, 10). Despite good prognostic value to these assessments (11), there has been no significant benefit from surgery and/or interventions based on these quantitative thresholds (12, 13).

It has become clear that the current standard of echocardiographic assessment, developed for primary MR, where the left heart has the advantage of intrinsic compliance (14) and time to compensate for volume overload (15), cannot be applied automatically to FMR without adjustments. There is emerging evidence that in this group of patients the volume loading from MR should be adjusted to the LV volume. A novel conceptual approach of using the ratio of MR effective regurgitant orifice (ERO) to left ventricular end-diastolic volume (LVEDV) has been suggested to explain differing outcomes in two recent, large, randomized, controlled trials of percutaneous mitral valve repair (16, 17) for patients with HF with significant FMR (18). There are ongoing calls for this approach to be validated in prospective studies (19). We have termed this value the proportionality index (PI).

The analysis of the implications of disproportionate MR has been investigated in individual retrospective assessments of both the MITRA-FR (20) and the COAPT (21) randomized-controlled trials alongside a combined appraisal (22). These assessments have provided conflicting results, with vigorous debate (23–25) and investigations as to the implications of disproportionality assessments based on either EROA/LVEDV > 0.14–0.15 or regurgitant volume (RV)/LVEDV > 0.2 (i.e., 20%) (26). This clearly calls for the assessment of this concept in a “real-world” clinical scenario faced by cardiologists and acute physicians.

Most previous studies have enrolled patients with *chronic* HF and optimized pharmacotherapy (17). Very little is known on the prevalence and significance of MR in patients presenting in *acute* HF. The handful of prospective studies investigating its role have not included either early or volumetric assessments and have mainly focused on stable patients (27, 28). Preliminary data from the European Heart Failure survey and US cohort studies suggest MR in hospitalized patients with HF is common (29, 30) but prognostic implications remain unclear. It is possible that MR is missed altogether in patients with AHF due to the dynamic nature of MR (31, 32), particularly if LV volumes remain within an accepted normal range. We, therefore, conducted a study to examine the prevalence and significance of MR in AHF and to determine whether proportionality indices would be effective at identifying patients who face adverse consequences of regurgitant mitral valves.

## Methods

### Patients and Trial Design

This was a prospective observational study to assess the prevalence of significant MR in consecutive patients admitted with an acute or exacerbation of chronic heart failure (A/ECHF) over 12 months following a 1 month rolling-in period in a single center [St Peter's Hospital (SPH), Chertsey, UK]. Enrolment, data collection, storage, and analysis occurred at this site. Hospital coding data from 2013 to 2016 was used to estimate a recruitment target of 500 patients.

Patients who displayed signs or symptoms of AHF were screened according to the pre-specified study protocol (Appendix 1 in Supplementary Material). Locations of assessment included the accident and emergency department, intensive care unit, high-dependency unit, acute medical unit, coronary care unit, respiratory ward, and care for the elderly ward. If A/ECHF was considered as the primary cause of admission following physician-led clinical examination, patients were consented and recruited into the study if bedside point-of-care brain natriuretic peptide (BNP) level was raised. They underwent transthoracic echocardiography (TTE) within 24 h of recruitment to assess cardiac and valvular function (Appendix 2 in Supplementary Material).

Patients with sepsis, respiratory failure secondary to pulmonary causes, stable chronic HF with an alternative diagnosis, and existing in-patients at the start of recruitment were not included. Patients in whom echocardiography was not possible (deceased, did not consent or discharged) were excluded from further analysis. All recruited patients were followed up for 2 years.

### Trial Oversight

The trial was designed by the physician-led executive committee in conjunction with Ashford & St Peter's Hospital Trust Research and Development team. The research protocol was approved by relevant institutional review boards and ethics committees and all participants gave written informed consent. The study complied with the Declaration of Helsinki.

Data were stored electronically and were available for review by all authors. The first and last authors developed the manuscript for submission. The design and implementation of this project and the decision to submit for publication were by the last author. Statistical analysis was carried out by an independent organization with established expertise in the statistical analysis including government policy projects.

### Study Data Collection

Diagnosis of AHF on admission was made by a dedicated study physician according to European Society of Cardiology (ESC) guidelines (10). BNP and TTE results were not disclosed to the emergency/acute clinical team. Demographic and past medical history data were identified from hospital records, while sex and ethnicity were self-reported by patients. Mortality data were recorded from the summary care record system used nationally by general practices in the United Kingdom and via the Evolve^TM^ (Kainos, United Kingdom) online software for in-patient deaths recorded by SPH. If unavailable, general practices and family members were directly contacted.

### Point-of-Care BNP

Point-of-care BNP measurement was performed using i-STAT Point of Care (POC) Serum BNP analyzer (Abbott, Illinois, USA) with cut-off value >100 pg/ml. This POC system has displayed good clinical agreement at lower BNP values (33). BNP cartridges were acquired and stored according to manufactures guidelines.

### Echocardiography

Echocardiography was performed using a dedicated G.E. Vivid S70 (GE Healthcare, Illinois, USA) machine. Images were stored and analyzed offline using EchoPac software version 201 (GE Healthcare, USA). Most of the TTE studies were performed by a single accredited operator according to study protocol (Appendix 3 in Supplementary Material). Every study was analyzed by the primary operator and cross-checked by an expert in echocardiography. Standard echo parameters of left heart geometry: (LVEDV and left ventricular end-systolic volumes (LVESV), LA area (LAA) were measured. MR quantitative analysis was performed using the PISA method to derive MR ERO area and regurgitant volume (RV) (34). Significant MR was defined as MR greater than mild severity, with grading categorized according to ESC guidelines (34). Systolic pulmonary artery pressure (sPAP) was estimated from tricuspid regurgitant jet and jugular vein respiratory fluctuations.

### Statistical Analysis

Data analysis was primarily carried out JB. Receiver Operator Curve analyses were carried out for the ERO and the PI (ERO/LVEDV). The optimum cut-off for the prediction of 24-month mortality was estimated by identifying the sensitivity and specificity associated with the maximum Youden Index. These cut-offs were then used as a binary determinant of proportionate vs. disproportionate MR. To evaluate volumetric assessments of proportionality in MR we also included the regurgitant volume/LVEDV and defined proportionate vs. disproportionate MR as < or > 0.2, as outlined in Namazi et al. (26).

Sociodemographic and baseline characteristics were summarized by severity group and overall for the complete analysis set. Categorical variables were reported as numbers and percentages and between-groups comparisons were compared using the chi-squared test or Fisher's exact test, as appropriate. Continuous variables were reported as means and standard deviations or as medians and interquartile ranges and compared using Student's *t*-test or the Mann–Whitney *U*-test.

For the primary analysis of 24-month mortality, unstratified Kaplan–Meier curves were constructed. Hazard ratios were estimated using an unadjusted Cox-regression model, with statistical significance being assessed using the log rank test. Secondary analyses were carried out using Cox-regression analyses adjusted for significant covariates. The selection of covariates to be included was based on initial multiple univariate regression analyses, modified according to clinical opinion from the research team. These were gender, age, body-mass index, and pre-existing diagnoses of chronic obstructive pulmonary disorder, hypertension, chronic kidney disease, ischemic heart disease, diabetes mellitus, and cerebrovascular disease. For all comparisons, the threshold of statistical significance was set at a two-sided α value of 0.05.

### Data Storage

Enrolled patients had an objective, echocardiographic and clinical characteristics collected *via* a standardized collection form which was stored online in a password-protected database specifically devised for study by Metanoic Health Ltd., United Kingdom.

Data were entered by primary operators and double-checked by independent specialists. Histograms were performed on all continuous data to screen for statistical outliers using Statistical Package for the Social Sciences (SPSS) version 24 (IBM, New York, USA). Any outlying data points were then rechecked to screen for input errors or errors of measurement. The echocardiography data was retained on two separate hard drives to allow for off-site analysis and to reduce the risk of data loss in accordance with Good Clinical Practice research protocols.

## Results

With a 1-month run-in period, 616 consecutive patients presenting with symptoms of A/ECHF were assessed for eligibility for the MRAHF study from July 2016 to August 2017. About 447 (72.6%) participants were recruited. About 418 individuals were included in the final analysis after excluding the data from rehospitalization and three individuals lost to follow-up.

All patients were found to have MR (100%) and 434/447 (97.1%) patients had functional MR as their underlying etiology. Based on clinical interpretation of MR on echocardiography patients were divided into two groups: all patients with moderate and above severity of MR were included in group 1 (significant MR) whereas all other patients in group 2 had mild MR. There was a high prevalence of ESC guideline-defined significant MR in our cohort. 165 (39.5%) of enrolled patients had significant MR, 253 (60.5%) had mild MR.

There were broad similarities in demographics, comorbidities, and presenting features between patients with significant and mild MR (Table 1). The mean age across both groups was 78.7; 53.1% were males and 93.3% self-identified as “white” ethnicity. Patients were highly symptomatic −361 (91.1%) with NYHA class III/IV presentation but not in cardiogenic shock [mean blood pressure (BP) 136/76 mmHg]. Patients did not have features of severe anemia or infection. The overall BNP averaged 1,363 ng/l and this was higher in patients with severe MR (1,729 ng/l) compared to mild MR (1,124 ng/l) (*p* < 0.0001).

**Table 1:** Baseline characteristics.

	**All patients (*n* = 418)**	**Significant MR (*n* = 165)**	**Mild MR (*n* = 253)**	*p*-value^*^
**Demographics**				
Age, mean (SD), y	78.7 (11.7)	79.3 (12.0)	78.3 (11.5)	0.395
Gender (male), n (%)	222 (53.1)	84 (50.9)	138 (54.6)	0.459
**Race**, ***n*** **(%)**				
White	390 (93.3)	150 (90.9)	240 (94.9)	0.110
BAME	28 (6.7)	15 (9·1)	13 (5.1)	0.110
BMI, mean: kg/m^2^ (sd)	28.6 (8.06)	29.5 (8.82)	27.2 (6.52)	0.004
**Comorbidities** ***n*** **(%)**				
Coronary artery disease	152 (36.4)	65 (39.4)	87 (34.4)	0.300
Hypertension	232 (55.5)	89 (53.9)	143 (56.5)	0.602
Diabetes	130 (31.1)	41 (24.9)	89 (35.2)	0.026
Chronic kidney disease	189 (45.2)	73 (44.2)	116 (45.9)	0.733
COPD	61 (14.6)	18 (10.9)	43 (17.0)	0.085
Cerebrovascular disease	64 (15.3)	30 (18.2)	34 (13.4)	0.183
**Presentation**				
NYHA class, *n* (%)				
II	37 (8.9)	12 (7.3)	25 (9.9)	0.361
III	161 (38.5)	61 (37.0)	100 (39.5)	0.608
IV	220 (52.6)	92 (55.8)	128 (50.6)	0.299
**ECG findings**				
Sinus rhythm, *n* (%)	163 (39.0)	56 (33.9)	107 (42.3)	0.086
AF, *n* (%)	192 (45.9)	85 (51.5)	107 (42.3)	0.065
Paced, *n* (%)	39 (9.3)	15 (9.1)	24 (9.5)	0.891
Other rhythm, *n* (%)	18 (4.3)	5 (3.0)	13 (5.1)	0.300
**Observations**				
BPs, mmHg mean (sd)	136 (26.4)	133 (25.4)	138 (27.0)	0.040
BPd, mmHg mean (sd)	76 (16.9)	75 (17.7)	76 (16.9)	0.539
HR, bpm mean (sd)	89 (27.2)	89 (27.7)	90 (26.9)	0.663
SpO_2_, % mean (sd)	95.0 (3.78)	95.2 (3.82)	94.8 (3.75)	0.209
**Biochemistry**				
Hemoglobin, g/l mean (sd)	122.5 (21.76)	121.6 (22.39)	123.1 (21.36)	0.486
Creatinine, μmol/l mean (sd)	120.0 (73.44)	126.9 (85.27)	115.6 (64.36)	0.148
eGFR, ml/min/1.73 m^2^ mean (sd)	48.3 (14.56)	47.1 (15.74)	49.1 (13.72)	0.181
CRP, mg/dl mean (sd)	29.5 (42.74)	31.9 (44.09)	28.0 (41.88)	0.385
BNP, ng/l mean (sd)	1,363 (1254.2)	1,729 (1315.7)	1,124 (1153.9)	<0.0001

*BAME, Black, Asian, and minority ethnic; COPD, chronic obstructive pulmonary disorder; NYHA, New York Heart Association; AF, Atrial fibrillation; BPs, blood pressure systolic; BPd, blood pressure diastolic; HR, heart rate; SpO_2_, peripheral capillary oxygen saturation; eGFR, estimated glomerular filtration rate; CRP, C-reactive protein; BNP, brain natriuretic peptide*.

* *p-values are estimated using Mann–Whitney U-test for medians, N−1 χ^2^ for proportions and independent samples t-test for continuous variables*.

The medical therapy at index admission was similar between both groups (Table 2) except for angiotensin-converting enzyme inhibitors/angiotensin receptor blockers (ACEi/ARBs) which were less common in the group with significant MR [34.5 vs. 46.6% (*p* = 0.032)]. Both groups had an increase in the intensity of HF therapy at discharge. There was a higher rate of prescription of mineralocorticoid receptor antagonists in the significant MR group [30.3 vs. 20.9% (*p* = 0.023)]. Based on this, the clinical team, blind to study findings, provided better optimization of medications for patients with significant MR.

**Table 2:** Medical therapy on admission and discharge.

	**Medications on admission**, ***n*** **(%)**			**Medications on discharge**, ***n*** **(%)**			Difference—discharge vs. admission^*^			
	**Significant MR (*n* = 165)**	**Mild MR (*n* = 253)**	*p* value^**^ (significant MR vs. mild MR)	**Significant MR (*n* = 165)**	**Mild MR (*n* = 253)**	*p* value^**^ (significant MR vs. mild MR)	**Significant MR**		**Mild MR**	
							***n* (% change)**	*p*-value^***^	***n* (% change)**	*p*-value^***^
ACEi/ARB	57 (34.5)	118 (46.6)	0.032	73 (44.2)	123 (48.6)	0.457	16 (10.1)	0.029	8 (3.7)	0.328
BB	91 (55.2)	140 (55.3)	0.865	126 (76.4)	184 (72.7)	0.252	41 (26.0)	<0.0001	47 (19.3)	<0.0001
MRA	26 (15.8)	25 (9.9)	0.081	50 (30.3)	53 (20.9)	0.023	26 (16.5)	<0.0001	28 (11.5)	<0.0001
Diuretic	89 (53.9)	118 (46.6)	0.180	135 (81.8)	191 (75.5)	0.053	51 (32.3)	<0.0001	75 (30.9)	<0.0001
CCB	27 (16.4)	50 (19.8)	0.352	17 (10.3)	44 (17.4)	0.051	−8 (−5.1)	0.134	−4 (−1.7)	0.652
Digoxin	21 (12.7)	28 (11.1)	0.638	28 (17.0)	54 (21.3)	0.304	9 (5.7)	0.108	26 (10.7)	<0.0001

*MR, mitral regurgitation; ACEi, angiotensin converting enzyme inhibitor; ARB, angiotensin receptor blocker; BB, beta-blocker; MRA, mineralocorticoid receptor blocker; CCB, calcium channel blocker*.

* *Results relate to 401 patients with paired admission/discharge data, so figures may vary from the difference of the values in the previous columns*.

** *Within-group p-values are estimated using N−1 χ^2^*.

*** *Between-group p-values are estimated using McNemar's test for paired proportions*.

Quantitative assessment of MR on echocardiography indicated significantly higher EROA and regurgitant volume (RV) in significant MR (Table 3). LV volumes remained within the normal range in both groups, however LVEDV [129.5 vs. 100.0 ml (*p* < 0.0001)] and LVESV [82.4 vs. 58.5 ml (*p* < 0.0001)] were greater in significant MR. The left atrium was also significantly larger [LAA 31.4 vs. 27.0 cm^2^ (*p* < 0.0001)]. LV ejection fraction (LVEF) differed between the groups [38.9 vs. 45.5% (*p* < 0.0001)] but remained above cut-off level for HF with reduced EF. The estimated sPAP was 57.2 mmHg in significant MR vs. 49.7 mmHg in mild MR [*p* < 0.0001]. Quantitative assessment was not performed in a minority of mild MR individuals due to insufficiency of jets. Trivial (<4%) numbers of LV/sPAP measurements were not obtained.

**Table 3:** Hemodynamic assessment by echocardiography.

	**All patients (*n* = 418)**	**Significant MR (*n* = 165)**	**Mild MR (*n* = 253)**	*p*-value^*^
**MR assessment**				
Qualitative assessment, *n* (%)				
Mild	253 (60.5)	0 (0.0)	253 (100.0)	–
Moderate	87 (20.8)	87 (52.7)	0 (0.0)	–
Severe	78 (18.7)	78 (47.3)	0 (0.0)	–
Quantitative assessment (QA)				
ERO, cm^2^; mean (SD)	0.23 (0.150)	0.33 (0.153)	0.14 (0.063)	<0.0001
QA not performed, *n* (%)	87 (20.8)	4 (2.4)	83 (32.8)	
RV, ml; mean (SD)	32.5 (20.14)	47.8 (17/79)	17.8 (7.10)	<0.0001
QA not performed, *n* (%)	88 (21.1)	4 (0.6)	84 (33.2)	
Vena contracta, mm; mean (SD)	0.38 (0.127)	0.47 (0.108)	0.30 (0.085)	<0.0001
QA not performed	60 (14.4)	1 (0.6)	59 (23.3)	
**Left-heart volumes and estimated systolic PA pressure**				
LVEDV, ml; mean (sd)	111.7 (55.5)	129.5 (58.95)	100.0 (49.91)	<0.0001
LVESV, ml; mean (sd)	68.0 (46.83)	82.4 (50.25)	58.5 (41.90)	<0.0001
EF, %; mean (sd)	42.9 (14.96)	38.9 (14.30)	45.5 (14.84)	<0.0001
LAA, cm^2^; mean (sd)	28.7 (8.21)	31.4 (8.47)	27.0 (7.56)	<0.0001
SPAP, mmHg; mean (sd)	52.7 (16.67)	57.2 (17.89)	49.7 (18.62)	<0.0001

*QA, quantitative assessment; QA not performed due to either insufficiency or complexity of MR jets. MR, mitral regurgitation; ERO, effective regurgitant orifice; RV, regurgitant volume; LVEDV, left ventricular end diastolic volume; LVESV, left ventricular end-systolic volume; EF, ejection fraction; LAS, left atrial size; SPAP, systolic pulmonary artery pressure; sd, standard deviation*.

* *p-values (significant MR vs. mild MR) are estimated using independent samples t-test*.

**Figure 1:**
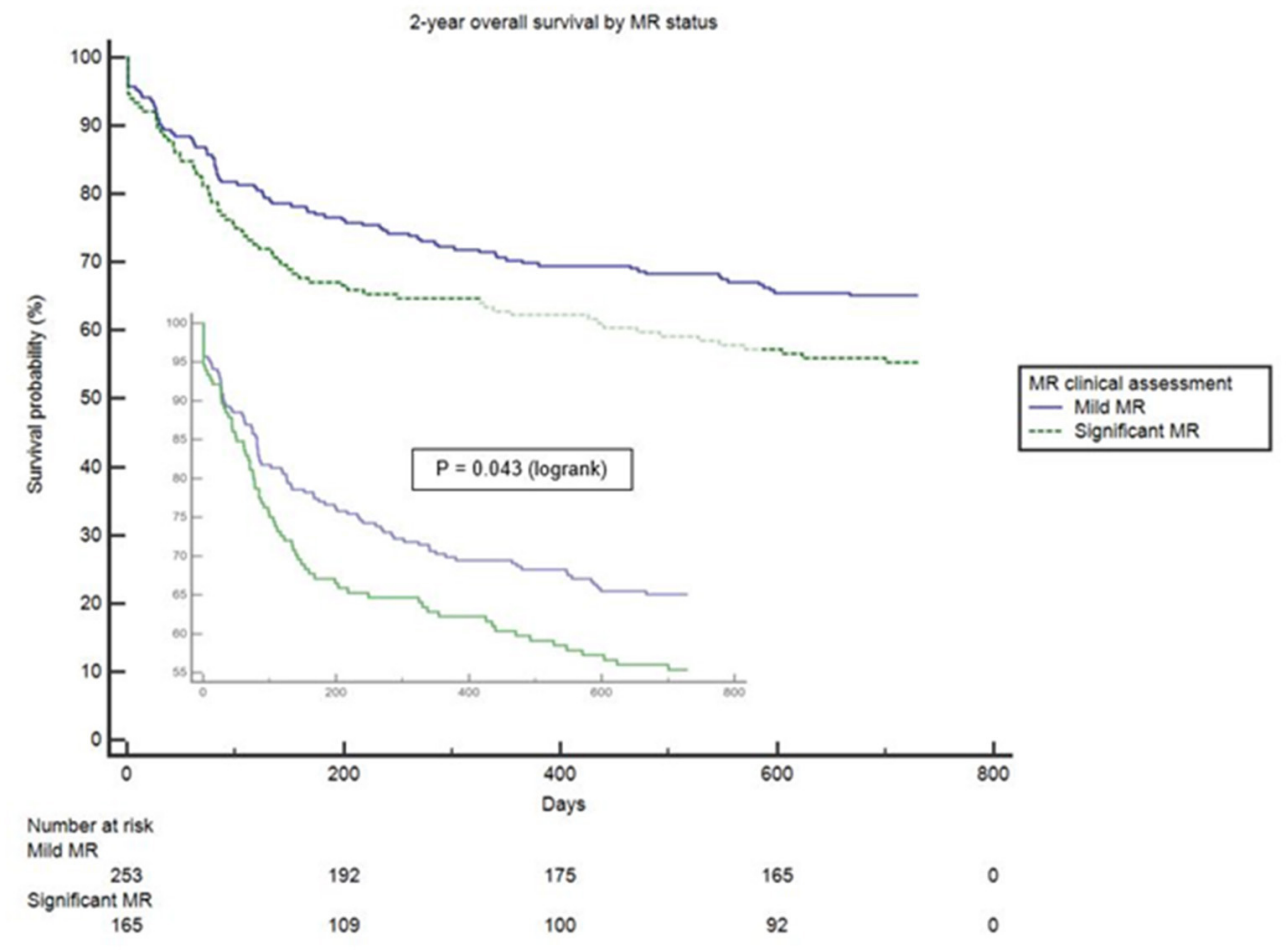
Unadjusted survival curve of 2-year all-cause mortality comparing mild and significant MR.

**Figure 2:**
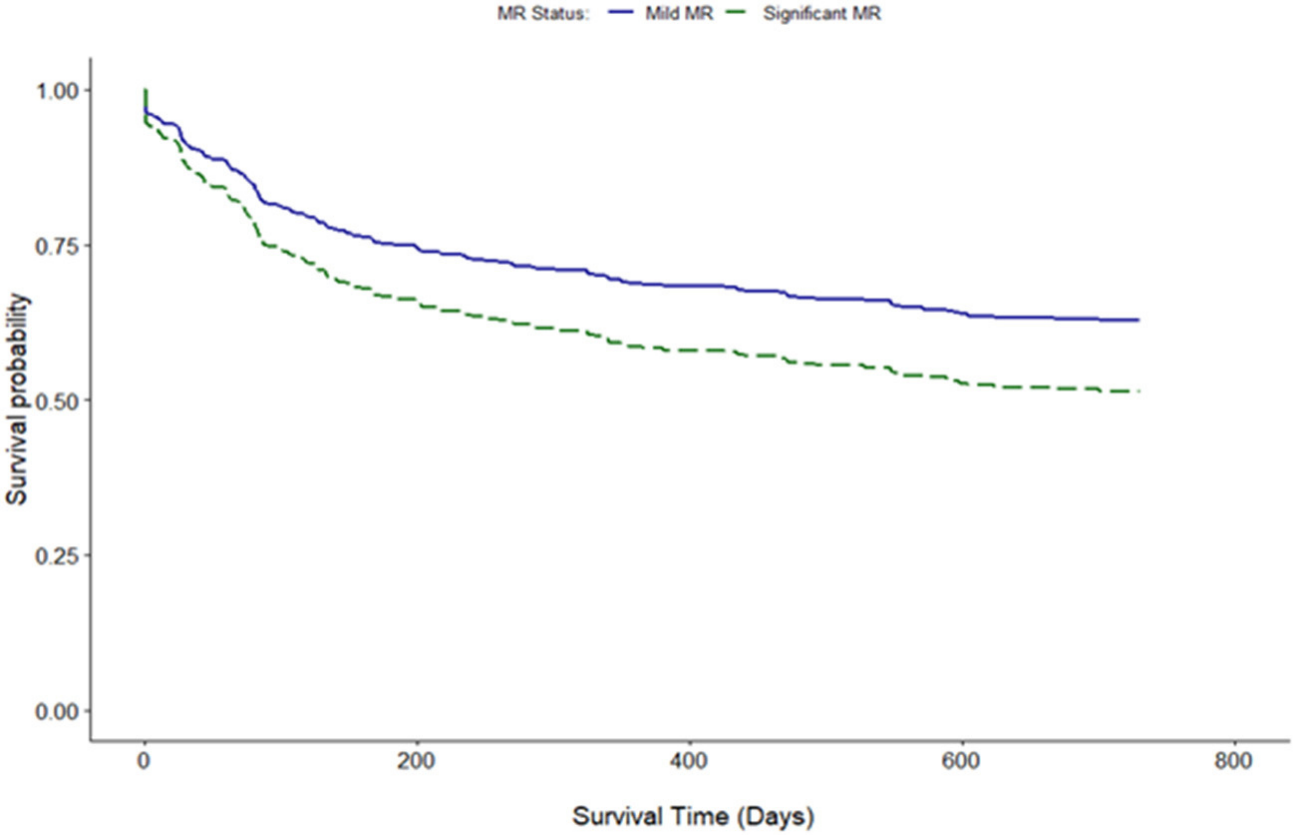
Adjusted survival curve of 2-year all-cause mortality comparing mild and significant MR.

**Figure 3:**
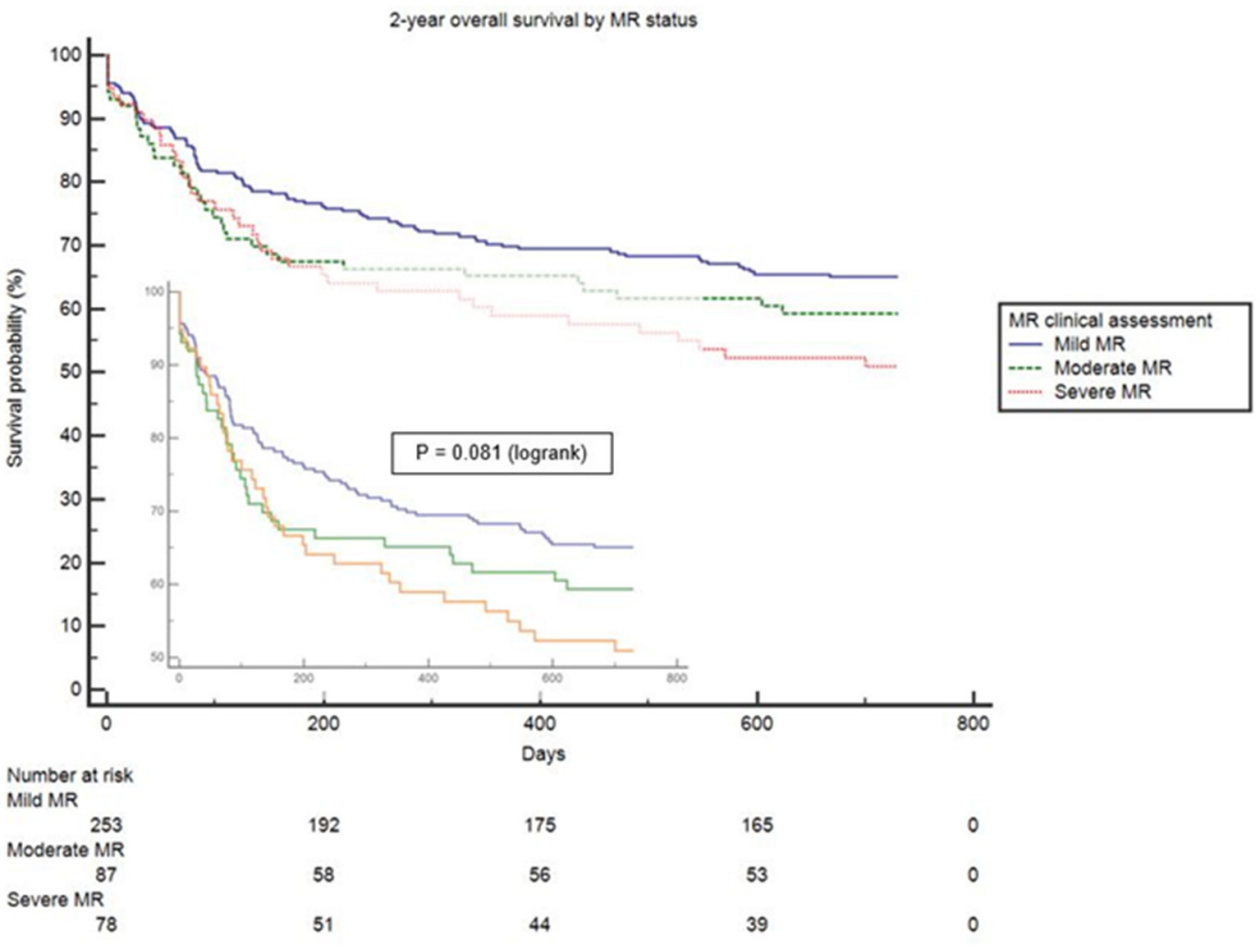
Survival curves of 2-year all cause mortality comparing mild, moderate and severe MR.

Clinical interpretation of significant MR was an important differentiator in the long-term outcome. At 2 years, those with significant MR had 73 (44.2%) deaths compared with 88 (34.8%) in the mild MR group (hazard ratio 1.39 [CI 1.01–1.92], *p* = 0.043) (Figure 1). Cox-regression analyses adjusted for multiple covariates confirmed that significant MR is associated with a greater risk of mortality at 2-years [hazard ratio 1.43 (1.04–1.97), *p* = 0.029] (Figure 2 and Table 4). Traditional echocardiographic grading of the severity of MR displayed a clear trend in survival but was not able to predict significant differences between the three severity grades (*p* = 0.081) (Figure 3 and Table 5).

**Table 4:** Multivariable Cox-regression analysis of MR defined by ERO/LVEDV > 0.14 mm^2^/ml.

**Predictive variables**	**HR for OS**	**95% CI**	***p*-value**
ERO to LVEDV ratio (Ratio > 0.14 cm^2^/ml)	1.54	[1·02, 2·34]	0·042
Gender–Male	1.04	[0.96, 0.72]	0.824
Age–Continuous	1.06	[1.03, 1.09]	<0.001
BMI–Continuous	0.99	[0.96, 1.02]	0.62
Known COPD–Yes	1.72	[1.07, 2.75]	0.024
Known hypertension -Yes	1.21	[0.83, 1.75]	0.326
Known CKD–Yes	1.81	[1.24, 2.63]	0.002
Known IHD–Yes	1.20	[0.83, 1.74]	0.329
Known diabetes–Yes	1.08	[0.71, 1.63]	0.723
Known cerebrovascular disease–Yes	0.74	[0.43, 1.27]	0.275

*MR, mitral regurgitation; ERO, effective regurgitant orifice; LVEDV, left ventricular end-diastolic volume; COPD, chronic obstructive pulmonary disorder; BMI, body-mass index; HTN, hypertension; CKD, chronic kidney disease; IHD, ischemic heart disease; DM, diabetes mellitus; CVD, cerebrovascular disease*.

**Table 5:** Kaplan–Meier estimates for overall survival at 24 months.

	**Clinical assessment of MR severity**		
	**Mild MR (*n* = 253)**	**Significant MR (*n* = 165)**	
Deaths–*N* (%)	88 (34.8)	73 (44.2)	
Data censored–*N* (%)	165 (65.2)	92 (55.8)	
Kaplan–Meier estimated OS* mean months (95% CI)	17.6 (16.4–18.7)	15.6 (14.1–17.2)	
Hazard ratio			
Significant vs. mild (95% CI)	1.39 (1.01–1.92)		
*p*-value (Logrank)	0.043		
	**Mild MR (*****n*** **=** **253)**	**Moderate MR (*****n*** **=** **87)**	**Severe MR (*****n*** **=** **78)**
Deaths–*N* (%)	88 (34.8)	35 (40.2)	38 (48.7)
Data censored–*N* (%)	165 (65.2)	52 (59.8)	40 (51.3)
Kaplan–Meier estimated OS* mean months (95% CI)	17.6 (16.4–18.7)	16.1 (13.9–18.2)	15.1 (12.9–17.4)
Hazard ratio			
Moderate vs. mild (95% CI)		1.25 (0.83–1.85)	
Severe vs. mild (95% CI)		1.51 (1.00–2.33)	
Severe vs. moderate (95% CI)		1.22 (0.74–2.04)	
*p*-value (Logrank)		0.081	
	**ERO/LVEDV assessment of MR severity**		
	**Mild (proportionate) MR (*****n*** **= 113)**	**Significant (disproportionate) MR (*****n*** **= 217)**	
Deaths–*N* (%)	32 (28.3)	92 (42.4)	
Data censored–*N* (%)	81 (71.7)	125 (57.6)	
Kaplan–Meier estimated OS* mean months (95% CI)	18.9 (17.4–20.5)	16.0 (14.7–17.4)	
Hazard ratio significant vs. mild (95% CI)	1.62 (1.12–2.34)		
*p*-value (Logrank)	0.0097		

*ERO, effective regurgitation orifice; LVEDV, left ventricular end-diastolic volume*.

**Figure 4:**
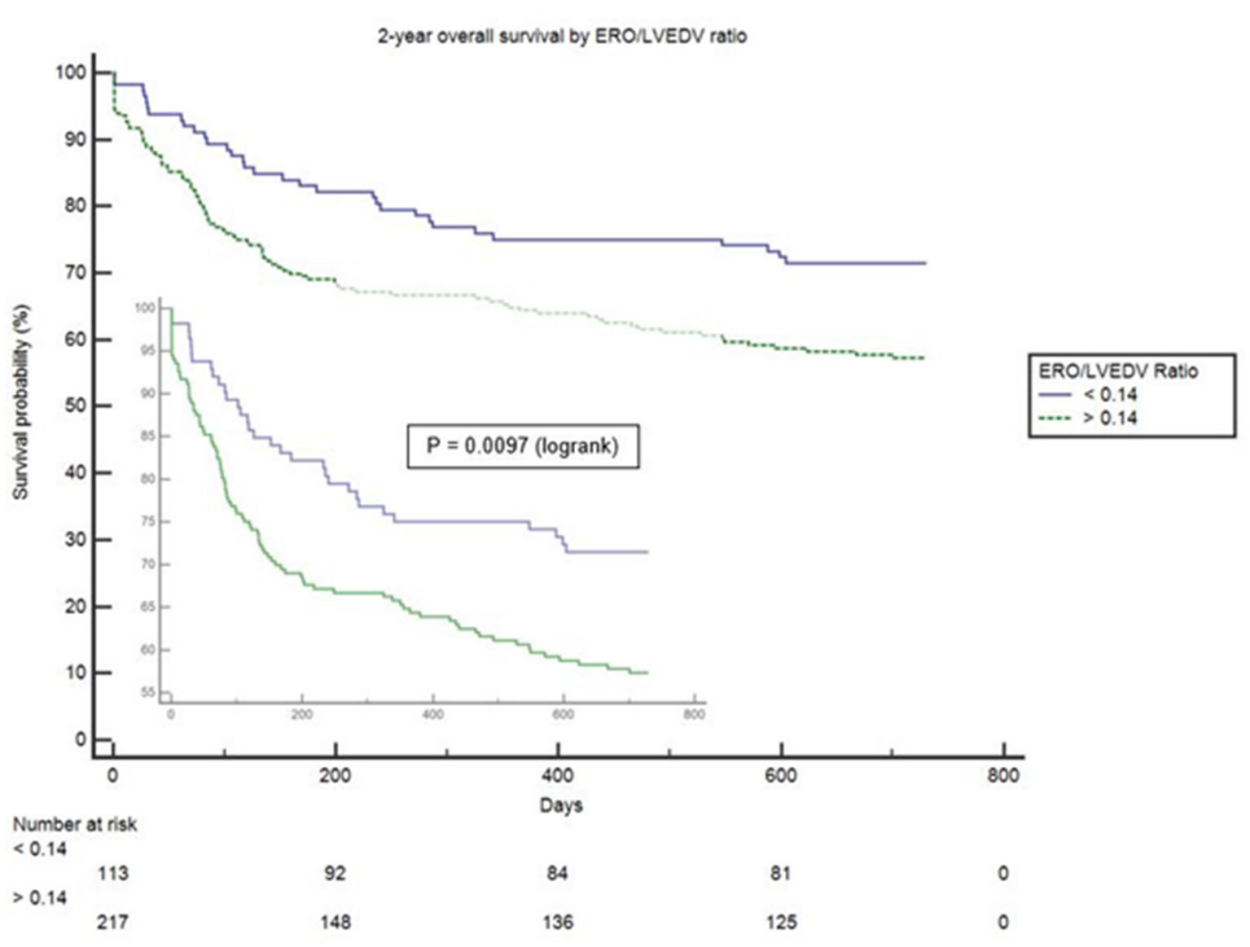
Unadjusted survival curve of 2-year all-cause mortality comparing proportionate and disproportionate MR, defined by an ERO/LVEDV ratio.

**Figure 5:**
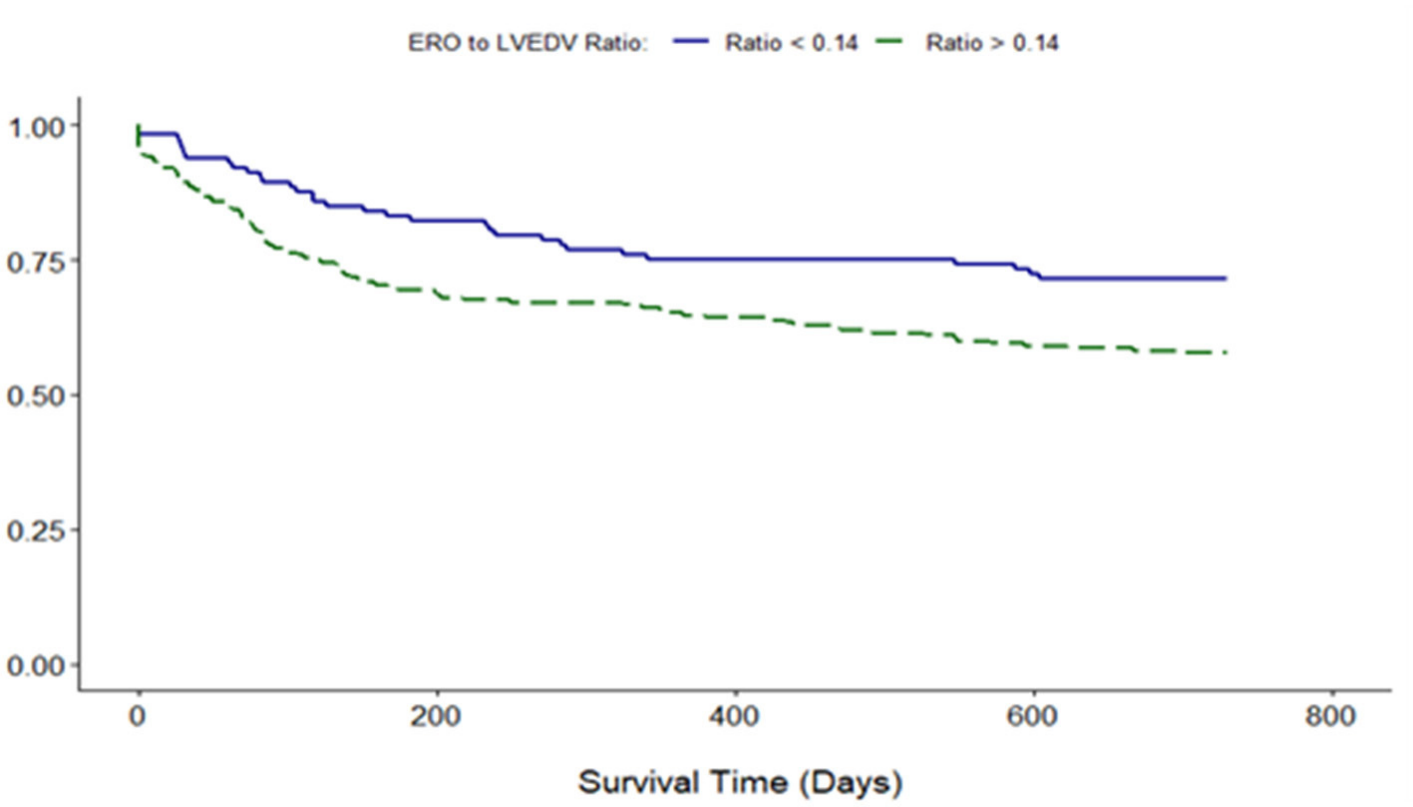
Adjusted survival curve of 2-year all-cause mortality comparing proportionate and disproportionate MR, defined by an ERO/LVEDV ratio.

Proportionality index (PI) cut-off was defined at 0.14 mm^2^/ml by ROC analysis. Disproportionate MR was discovered in 217/331 individuals (65.6%). Regardless of the magnitude of volume overload, the presence of disproportionate MR was an important predictor of outcome from index event; there were 92 (42.4%) deaths compared with 32 (28.3%) in patients with and without proportionate MR [hazard ratio (HR) 1.62 (CI 1.12–2.34), *p* = 0.010] (Figure 4 and Table 5). Cox-regression analyses adjusted for multiple covariates also confirmed that disproportionate MR is associated with a greater risk of mortality at 2 years [HR 1.54 (1.02–2.34), *p* = 0.042] (Figure 5 and Table 6). Volumetric disproportionate MR (defined by RV/LVEDV > 0.2) was discovered similarly in 222/345 (64.3%) patients. There were 95 (42.8%) deaths in patients with disproportionate MR defined by regurgitant volumes, significantly more than the 39 (31.7%) with proportionate MR (*p* = 0.045).

**Table 6:** Multivariable Cox-regression analysis of significant MR for overall survival at 24 months.

**Predictive variables**	**HR for OS**	**95% CI**	***p*-value**
Significant MR	1.43	[1.04, 1.97]	0.029
Gender	0.97	[0.70, 1.34]	0.9
Age	1.05	[1.03, 1.07]	<0.001
BMI	0.99	[0.96, 1.01]	0.2
Known COPD	2.08	[1.40, 3.08]	<0.001
Known HTN	1.20	[0.87, 1.67]	0.3
Known CKD	1.76	[1.26, 2.45]	<0.001
Known IHD	1.05	[0.76, 1.46]	0.8
Known DM	1.16	[0.81, 1.65]	0.4
Known CVD	0.84	[0.53, 1.32]	0.4

*MR, mitral regurgitation; BMI, body mass index; COPD, chronic obstructive pulmonary disorder; HTN, hypertension; CKD, chronic kidney disease; IHD, ischemic heart disease; DM, diabetes mellitus; CVD, cerebrovascular disease*.

## Discussion

This is the first “real-world” prospective study to assess the prevalence of MR in patients presenting with *acute* HF to an emergency department before the effect of intensive diuresis. In contrast to previous studies (30), patients with HF presenting with sepsis and other medical emergencies were excluded. Our study revealed that all patients requiring admission had some degree of MR. There was a high prevalence of traditionally defined clinically significant MR of moderate to a severe degree (39.5%), and disproportionate, MR defined by an index of proportionality defined by the ERO/LVEDV > 0.14 (65.6%).

Demographic and other clinical characteristics remained broadly similar between those presenting with significant and mild MR. BNP, a well-established biomarker of ventricular disease severity in degenerative and functional MR (35), was the only distinguishing clinical parameter between patients with and without significant MR. However, there is no clear cut-off level for its use in AHF due to the heterogeneous nature of the myocardial injury. We, therefore, used portable, bedside echocardiography to identify and quantify MR. This was particularly important given that functional MR tends to be dynamic in nature and will likely settle with aggressive diuresis. Dynamic MR has been proven to have a prognostic impact in AHF (28) and we have expanded *early* hemodynamic assessment further by using volume-indexed parameters of MR.

Functional mitral regurgitation (FMR) is a distinct entity in terms of pathophysiology and prognostic implications (36) and to our knowledge, there is no consensus as to the timing of hemodynamic assessment of MR in AHF. Prior studies investigating MR in HF often require optimization of medical therapy before enrolment (17). Moreover, the severity and stage of underlying left heart geometric change due to ischemia, progressive myocardial disease, and/or LA enlargement makes it difficult to have a uniform approach for the assessment of left heart geometry (37, 38). MR is considered to be severe when the volume of chronic MR expands LV size beyond a given threshold—which has previously been determined to have a prognostic impact (10, 11). In our study, LV volumes were larger in patients with significant MR but remained within the normal range and significantly lower than in patients enrolled in percutaneous intervention trials for MR (16, 17).

The observed 2-year mortality in our cohort (38.5%) did not substantially differ from other AHF studies (1). The differences in short- and long-term mortality remained significant even after multivariate adjustments for comorbid conditions and demographics. Standard echo assessment (32) was useful but did not provide a clear separation point in survival between moderate and severe MR beyond clinical evaluation. Despite the absence of severe LV remodeling, patients with significant MR had higher mortality rates compared to the mild MR group despite similar (if not better) optimization of pharmacotherapy on index admission. At our center discharge medications suggested more intensive HF therapy in patients with significant MR.

When MR EROA was adjusted to LV volumes using a PI > 0.14 mm^2^/ml we observed a rapid separation in survival from index admission for patients with disproportionate MR. We did not observe differences in either the prevalence or prognostic implications of using the EROA indexed to LVEDV as compared to the MR regurgitant volume.

Our study indicates that hearts that are disproportionately affected by MR carry a greater risk of mortality, suggesting MR is an active driver of poor outcomes. Our study suggests either the EROA or RV is a clinically useful indexing parameter in the context of AHF. Subject to further confirmation by other outcome studies, our data asserts that functional MR should be assessed and managed completely differently to primary MR—using adjustments, namely, ratio/indexed parameters, rather than absolute volumetric analysis, to define thresholds for intervention in FMR patients.

The differences in the pathophysiology between primary and secondary MR (including the rate of change of atrial compliance) should therefore predicate adjustment of echocardiographic evaluation of regurgitant jets, transvalvular flow, the subvalvular apparatus, and the ventricle itself. We suggest that the current standards of cardiac assessment in HF should be updated to reflect the findings from this study and to lower the threshold of LV volumes for prognostically significant MR. This approach to the assessment of functional MR might become an important additional predictive tool to the current biomarkers such as BNP and cardiac troponin (39). This would be of particular benefit to individuals who could undergo surgical/catheter-based interventions to correct FMR.

The strengths of our study include the long-term follow-up, the consecutive enrolment of AHF presentations, and the small number of patients lost to follow-up. A limitation of our study is that it was undertaken at a single center where a majority of our patients self-identified as “White” ethnicity. However, interoperator variability in TTE is a well-characterized limitation of echocardiography and the single-center design of our study facilitated the use of a single operator in most echo assessments in our study, mitigating this limitation. We also did not adjust for differences between treatments in our groups because both admission pharmacotherapy and optimization at discharge occurred similarly between groups according to local and national guidelines. We assume that the difference in mortality would have been broader given more intensive HF therapy in patients with significant MR.

In conclusion, our prospective study demonstrated the high mortality of patients presenting in AHF, particularly those complicated by disproportionate MR. This approach of rapid MR evaluation might help identify those patients likely to benefit from interventions beyond pharmacological optimization. We consider these findings a significant “real-world” addition = to the ongoing debate on the management of disproportionate MR which has direct relevance to both acute physicians and cardiologists. Subject to further confirmatory studies, MR, particularly disproportionate, should not be ignored as a reflection of underlying poor LV performance but viewed as an active driver of poor outcome.

## Data Availability Statement

The raw data supporting the conclusions of this article will be made available by the authors, without undue reservation. Anonymised/deidentified data will be made available for a period of 6 months from the publication of this article and made available at request with a signed data access agreement. Study protocol and statistical analysis plan will be made available from publication for a period of 6 months.

## Ethics Statement

The studies involving human participants were reviewed and approved by Ashford and Saint Peter's NHS Foundation Trust. The patients/participants provided their written informed consent to participate in this study.

## Author Contributions

MB and AB developed the manuscript for publication. AB is a principal investigator and designed the concept. IB, DF, IJ, PS, and AB planned the study protocol. OL and EA were primarily responsible for echocardiographic data collection and analysis, supervised by AB. JS, JB, IJ, and MB were responsible for patient recruitment, database curation, and verifying the data along with the main statistical analysis which was implemented and designed by JB with clinical input from MB and AB. All authors had access to the database and all authors reviewed the manuscript before publication.

## Funding

This study was funded by ASPH R&D and Abbott Laboratories. They had no role in the study design, data collection, data analysis, data interpretation, or writing of the report. AB had final responsibility for the decision to submit for publication.

## Conflict of Interest

JB is owner of the company JB Medical Ltd, an independent statistical company which was not involved in the funding of this study. The remaining authors declare that the research was conducted in the absence of any commercial or financial relationships that could be construed as a potential conflict of interest.

## Publisher's Note

All claims expressed in this article are solely those of the authors and do not necessarily represent those of their affiliated organizations, or those of the publisher, the editors and the reviewers. Any product that may be evaluated in this article, or claim that may be made by its manufacturer, is not guaranteed or endorsed by the publisher.
