# Supplementary material for: Disproportionate Mitral Regurgitation Determines Survival in Acute Heart Failure
Source: Front Cardiovasc Med. 2021 Dec 2;8:742224. doi: 10.3389/fcvm.2021.742224 (PMC8675886; doi:10.3389/fcvm.2021.742224)
Supplement: Supplementary file 1 [file Data_Sheet_1.docx]

- **Appendix 1**

MRAHF study protocol

| Patients admitted to ITU, HDU, Acute Medical Unit (AMU), coronary care unit (CCU), respiratory ward and care for elderly ward with pulmonary oedema, shortness of breath, clinical features of left and right heart congestion and/or palpitations.  Shortness of Breath (SOB), peripheral oedema, arrhythmias (AF, SVT, VT, Frequent VEs)  ꜜ ꜜ  No Yes  ꜜ ꜜ  No Further Action Clinical Examination and Phonocardiography  Systolic Murmur,  ꜜ ꜜ  Yes No  ꜜ ꜜ  i-STAT BNP i-STAT BNP  ꜜ ꜜ ꜜ ꜜ  Normal Elevated Elevated Normal  $\downarrow$ $\downarrow$ $\downarrow$ $\downarrow$  No Further TTE TTE No Further  Action $\downarrow$ $\downarrow$ Action  Grading of Mitral Regurgitation on TTE  $\downarrow$ $\downarrow$ $\downarrow$  0-2 3 4  $\downarrow$ $\downarrow$ $\downarrow$  No Further Action Retrospective Analysis of Notes |
| --- |

**Appendix 2**


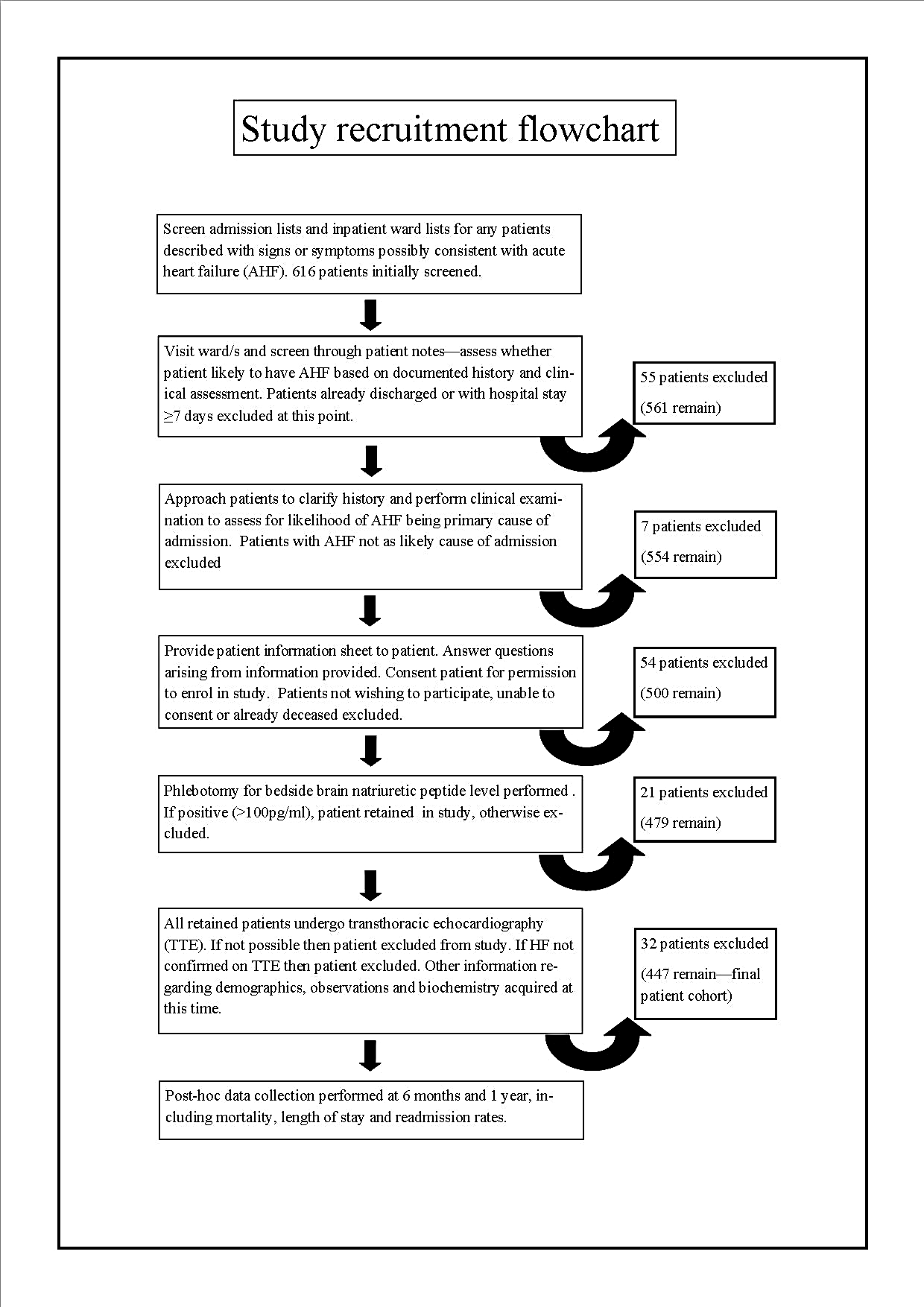


**Appendix 3**

Echocardiographic assessment protocol.

*Views and acquisition requirements*

*(2 cardiac cycles in sinus rhythm, 5 cardiac cycles in atrial fibrillation)*

1. *Parasternal Long Axis view:*

• 2D loop at moderate (50-70/sec) frame rate

• 2D loop with colour Doppler

• 2D loop with colour tissue Doppler at high (>120/sec) frame rate

• M-mode (still frame) of aortic root/left atrium

• M-mode (still frame) of left ventricle

• 2D loop (zoomed) of aortic root for Left-ventricular outflow tract dimension

• 2D loop RV inflow view (optional)

• 2D loop RV inflow view with colour wave Doppler for TR (optional)

1. *Parasternal Short Axis view:*

• 2D loop at aortic valve level

• Pulse wave Doppler of right ventricular outflow tract (sweep)

• 2D loop of MV in short axis

• 2D loop with colour Doppler at MV level at high frame rate

• 2D loop at papillary muscle level at moderate frame rate

• 2D loop with colour tissue Doppler at high (>120/sec) frame rate

• 2D loop at mid left ventricular level

• 2D loop at apical left ventricular level

1. *Apical Four Chamber View:*

• 2D loop at moderate frame rate focusing on all 4 chambers

• 2D loop with colour flow Doppler on all 4 chambers

• 2D loop zoom at moderate frame rate focusing on left and right ventricles

• 2D loop zoom with colour tissue Doppler at high frame rate focusing on left and right ventricles

• Pulsed tissue Doppler of the lateral mitral annulus.

• Pulsed tissue Doppler of the septal mitral annulus

• Pulsed tissue Doppler of the right ventricular free wall annulus

• 2D loop with colour flow Doppler focusing on MR (must see proximal isovelocity surface area (PISA), vena contracta (VC) and jet in left atrium)

• Zoomed 2D loop with colour flow Doppler focusing on MR (must see PISA, VC and

jet in LA)

• 2D loop with colour flow Doppler focusing on tricuspid regurgitation (TR)

• 2D loop at high frame rate to include both atria and pulmonary veins

• Pulsed Doppler of the mitral inflow (sweep)

• Pulsed wave Doppler of the right upper pulmonary vein (sweep)

• Continuous wave Doppler of the MR jet

• Pulsed Doppler of the left ventricular outflow (sweep)

• Continuous wave Doppler of aortic outflow

• Pulsed Doppler of the tricuspid inflow (sweep)

• Continuous wave Doppler of the TR jet

1. *Apical Two Chamber view:*

• 2D loop at moderate frame rate (LA/LV)

• 2D loop with colour flow Doppler

• 2D loop at moderate frame rate focusing on left ventricle

• 2D loop with colour tissue Doppler at high frame rate focusing on left ventricle

• 2D loop zoom at high frame rate on left atrium and pulmonary veins

• 2D loop with colour flow Doppler focusing on MR (must see PISA, VC and jet in LA)

• Zoomed 2D loop with colour flow Doppler focusing on MR (must see PISA, VC and

jet in LA)

1. *Apical Long Axis view:*

• 2D loop at moderate frame rate

• 2D loop at moderate frame rate focusing on left ventricle

• 2D loop with colour Doppler

• 2D loop with colour flow Doppler focusing on MR (must see PISA, VC and jet in LA)

• Zoomed 2D loop with colour flow Doppler focusing on MR (must see PISA, VC and

jet in LA)

1. *Subcostal view:*

• Inferior vena cava dimension and sniff test – 2D ± M-mode

• 2D loop of Subcostal long axis view

• 2D loop of Subcostal short axis view at papillary muscle level (optional)

• 2D loop of Subcostal short axis view at atrioventricular level (optional)
